# Extensive colonization with carbapenemase-producing microorganisms in Romanian burn patients: infectious consequences from the Colectiv fire disaster

**DOI:** 10.1007/s10096-017-3118-1

**Published:** 2017-10-23

**Authors:** L. E. Pirii, A. W. Friedrich, J. W.A. Rossen, W. Vogels, G. I. J. M. Beerthuizen, M. K. Nieuwenhuis, A. M. D. Kooistra-Smid, E. Bathoorn

**Affiliations:** 1 Department of Medical Microbiology, University of Groningen, University Medical Center Groningen, Groningen, The Netherlands; 2 Department of Medical Microbiology, Certe, Groningen, The Netherlands; 3 Department of Medical Microbiology, Martini Hospital, Groningen, The Netherlands; 4 Burn Centre Martini Hospital, Groningen, The Netherlands; 5 Association of Dutch Burn Centers, Burn Centre, Martini Hospital Groningen, Groningen, The Netherlands

**Keywords:** Carbapenemase, CPE, Burn patients, Infection control, Review, Molecular epidemiology

## Abstract

Health care of severe burn patients is highly specialized and may require international patient transfer. Burn patients have an increased risk of developing infections. Patients that have been hospitalized in countries where carbapenemase-producing microorganisms (CPMO) are endemic may develop infections that are difficult to treat. In addition, there is a risk on outbreaks with CPMOs in burn centers. This study underlines that burn patients may extensively be colonized with CPMOs, and it provides best practice recommendations regarding clinical microbiology and infection control. We evaluated CPMO-carriage and wound colonization in a burn patient initially treated in Romania, and transported to the Netherlands. The sequence types and acquired beta-lactamase genes of highly-resistant microorganisms were derived from next generation sequencing data. Next, we searched literature for reports on CPMOs in burn patients. Five different carbapenemase-producing isolates were cultured: two unrelated OXA-48-producing *Klebsiella pneumoniae* isolates, OXA-23-producing *Acinetobacter baumanii*, OXA-48-producing *Enterobacter cloacae*, and NDM-1-producing *Providencia stuartii*. Also, multi-drug resistant *Pseudomonas aeruginosa* isolates were detected. Among the sampling sites, there was high variety in CPMOs. We found 46 reports on CPMOs in burn patients. We listed the epidemiology of CPMOs by country of initial treatment, and summarized recommendations for care of these patients based on these reports and our study.

## Introduction

In October 2015, the crowded nightclub Colectiv in Bucharest, Romania caught on fire due to indoor use of pyrotechnics. In total, 64 visitors died from burn wounds and/or inhalation of smoke, and 144 were injured. The injured visitors were immediately transported to 12 nearby hospitals in Bucharest and Ilfov County for medical care [1]. Since appropriate medical care could not be provided for all patients, international aid was requested. About 80 patients were transported to various countries, including 16 to The Netherlands and Belgium, after they had been hospitalized for over a week in Romania.

Romania is a country with a high prevalence of carbapenemase-producing microorganisms (CPMO) [2, 3]. As burn patients have a high risk of developing infectious complications, this is a serious problem to be reckoned with. Wound infection with CPMO complicates the treatment of patients with burns [4, 5]. Patients suffering from these kind of infections have to be treated with last-line antibiotic schemes. These schemes are most often sub-optimal for treatment of the infection and have more adverse effects. In addition to the impact of CPMO-infection on the treatment of the individual patient, the introduction of CPMOs in the hospital also may lead to nosocomial transmission of CPMO resulting in hospital outbreaks.

Burn wounds are highly prone to long-term colonization by nosocomial bacteria. It has been reported that in more than 90% of patients the wounds were colonized by the seventh day, and that constitution of colonizing microorganisms in individual burn wounds changes over time [6, 7]. Wound colonization can subsequently result in severe invasive infection, a leading cause of mortality in patients with burn injury [8].

Restrictive and targeted use of antibiotics is important in treatment of burn patients, in particular in those with CPMOs. Guidelines from the European Burns Association recommend the use of “topical creams with good antimicrobial effects without the risk for resistance or allergy”. “The use of prophylactic systemic antibiotics is not supported by evidence” [9, 10]. Infections are most often caused by the microorganisms that colonize the burn wounds [11, 12]. Thus, it is important to culture wounds on admission, also before signs of infection, to know which antibiotics to start in case of infection.

Here, we describe the diversity in CPMO cultured at admission from several burn wounds and body sites in a burn patient from the Colectiv fire disaster transported to a dedicated Burn Centre in the Netherlands. Next, we performed an analysis of literature focusing on CPMO in patients with burns. By this, we show that the presented case of extensive burn wound colonization with CPMO is not an exception. Finally, we provide specific recommendations for medical care of burn patients transported from CPMO endemic regions to other countries with low CPMO prevalence. For non-endemic countries such as the Netherlands, international transfer of patients carrying CPMOs imposes a risk on dissemination to other hospitalized patients.

## Case description

A Romanian victim of the Colectiv fire disaster had been admitted to “Spitalul Clinic de Urgenta” in Bucharest on October 31st 2015 with a total body surface area (TBSA) burn of approximately 30%. The patient was in his 20s and had an uncomplicated medical history before this incident.

There were IIA–IIB degree burn lesions on the face, posterior cervical area, right scapular area, deltoid area bilaterally and IIB–III degree burns on both hands, forearms, and scalp. Meshed split skin grafting had been performed to cover the burns on his right lower arm and hand. On the IC unit, the patient had received broad-spectrum empirical antibiotic therapy with Piperacillin/ Tazobactam 4.5 g tid and Linezolid 600 mg bid. Based on results of wound cultures that revealed *Acinetobacter spp.*, antibiotics were switched to Colistin 2 million units tid for the treatment of wound infection. For topical treatment of the wounds, silversulfadiazine, kanamycine ointments, and betadine scrub were used. No additional information on the microbiological cultures was mentioned in the Romanian discharge notes.

The patient was transported by airplane and ambulance to the Burn Centre of the Martini Hospital (BCMH) in Groningen, The Netherlands on November 7th 2015, 7 days after the incident. Upon admission to the BCMH, the patient’s TBSA burned was still approximately 10%. Admission cultures taken from the wounds and body sites (nose, throat, perineum) showed extensive colonization with CPMOs (see results section). Following regular Dutch infection control recommendations, the patient was consequently placed in isolation. However, in this phase there was no need for treatment with systemic antibiotics. The burn wounds were topically treated with silversulfadiazine ointment. After 12 days, definitive covering of non-healing sites was opportune after enlargement of autologous donor skin in a ratio of 1:1.5. Skin defects on both hands and ears were covered with skin grafts taken from the right upper leg. Good take of the grafts was observed in the weeks after surgery. Pressure gloves were used to augment the healing of the hands. Through extensive physical and occupational therapy, the patient regained his ability to perform normal daily activities. The patient was discharged from the hospital after 34 days.

After discharge, the patient’s air-locked room with sanitary facility was disinfected. Subsequently taken environmental samples were negative.

## Methods

### Culture and characterization of bacterial isolates

Upon admission, screening throat, nose, perineum, rectum, and wound sample cultures were taken for detection of MRSA and highly-resistant gram negative bacteria (HRGN). Cultures from wounds were taken from the following locations: the anterior left elbow, the left and right ear, right shoulder and the left groin on November 9th; the left palm, and right upper back side on November 16th; the dorsum of the left hand, the right fingers and a repeated culture of right and left ear on November 30th. In total, 29 cultures were taken during the hospital stay: 14 screening cultures, three urine cultures, and 12 wound cultures. Burn wounds were cultured using sets of RODAC plates with five different media: blood agar +**5**% sheepblood (BA + 5%SB), colistin oxolinic-acid blood agar, mannitol salt agar, MacConkey agar no.3 + crystalviolet, Sabouraud dextrose agar + aztreonam/vancomycin (Mediaproducts, the Netherlands). For sampling, the plates were applied directly on the wounds. The plates were incubated for 48 h at 35 °C. Screening for methicillin-resistant *Staphylococcus aureus* (MRSA) was done with Xpert MRSA Gen3 assay (Cepheid, France) and by culture using BA + 5%SB and CHROMagar ID MRSA (bioMérieux, France) plates. Species determination of isolates was performed by using Maldi-TOF MS (bioMérieux, France). Antibiotic susceptibility was tested using VITEK 2 XL (bioMérieux, France). Minimal inhibitory concentrations (MICs) to tigecyclin, amikacin, and fosfomycin were tested using Etests according to manufacturer’s guidelines on Mueller Hinton agar (AB Biodisk, Germany). Susceptibility was interpreted according to EUCAST guidelines [13]. Using whole genome sequencing data, we characterized the CPMO isolates and identified acquired resistance genes as described before [14]. In short, genomic DNA was extracted and prepared libraries were run on a MiSeq platform (Illumina, USA) generating paired-end 250-bp reads. De novo assembly of paired-end reads was performed using CLC Genomics Workbench v7.5 (QIAGEN, Germany) after quality trimming (Qs ≥ 20) with optimal word size. The acquired antimicrobial resistance genes were identified by uploading assembled genomes to the Resfinder server v2.1 [15]. The MLST-types were assessed using SeqSphere v3.4.0 (Ridom GmbH, Germany).

Patient informed consent and approval of local ethical committee have been obtained. All of the assessed culture samples were taken in routine diagnostics.

### Literature analysis

We performed a literature search in PubMed to assess the epidemiology of CPMOs in burn wound care and recommendations for care of these patients by the following search strategy: ((burn[MeSH] OR burn*[TIAB] OR burn*[All Fields])) AND ((carbapenemase[MeSH] OR carbapenemase[All Fields] OR carbapenem resistant[MeSH] OR carbapenem resistant[All Fields] OR carbapenemase producing organisms[MeSH] OR carbapenemase producing organisms[All Fields] OR carbapenemase producing Enterobacteriacae[MeSH] OR carbapenemase producing Enterobacteriacae[All Fields] OR panresistant[MeSH] OR panresistant[All Fields] OR carbapenemase producing microorganisms[MeSH] OR carbapenemase producing microorganisms[All Fields])). Studies up to December 2016 were retrieved and screened by their title and abstract for their relevancy on the topic.

## Results

### Cultures

MRSA diagnostics were all negative; methicillin-susceptible *S. aureus* was cultured from nose and the burn wounds. We present an overview of characteristics of the isolated HRGNs in Table 1. In total, six different HRGNs were detected: five different carbapenemase-producing isolates, and one carbapenem-resistant *Pseudomonas aeruginosa* isolate. The carbapenemase-producing isolates included OXA-48-producing *Klebsiella pneumoniae* isolates of ST type 147, and 395, OXA-23-producing *Acinetobacter baumanii* ST type 231, OXA-48-producing *Enterobacter cloacae* ST type 114, and NDM-1-producing *Providencia stuartii*.

**Table 1:** Characteristics of the isolated highly-resistant Gram negative bacteria (HRGNs)

Isolate	Date	Sample	MIC MER	MIC IMP	Sensitivity					MIC COL	MIC TIG	*Bla*-genes^a^	ST type
			(mg/L)	(mg/L)	AK	GN	SXT	CIP	FOS	(mg/L)	(mg/L)		
***A. baumanii***	30–11-2015	Left ear	> = 16 R	> = 16 R	R	R	R	R	R	<=0.5 S	3 R	OXA-23; OXA-64	ST 231
***E. cloacae***	7–11-2015	Perineum	8 I	> = 16 R	S	R	R	R	R	<0.5 S	2 R	OXA-48; CTX-M 15; OXA-1; TEM-1b; ACT-16	ST 114
***K. pneumoniae***	9–11-2015	Right ear	> = 16 R	> = 16 R	R	R	R	R	S	> = 16 R	3 S	OXA-48; CTX-M-15; OXA-1; NDM-1; TEM-1b	ST 147
***K. pneumoniae***	7–11-2015	Perineum	> = 16 R	> = 16 R	S	R	R	R	R	<=0.5 S	1.5 S	OXA-48; CTX-M 15; OXA-1; TEM-1b; SHV-11	ST 395
***K. pneumoniae***	30–11-2015	Perineum	> = 16 R	> = 16 R	S	R	R	R	R	<=0.5 S	1,5 S	OXA-48; CTX-M-15; OXA-1; TEM-1b; SHV-11	ST 395
***K. pneumoniae***	7–11-2015	Rectum	>32 R	12 R	S	R	R	R	R	<=0.5 S	2 R	OXA-48; CTX-M 15; OXA-1; TEM-1b; SHV-11	ST 395
***P. stuartii***	9–11-2015	Left groin	> = 16 R	> = 16 R	R	R	R	R	S	>16 R	3 R	NDM-1; OXA-10; CMY-4	n.a.
***P. stuartii***	23–11-2015	Perineum	> = 16 R	> = 16 R	R	R	R	R	S	> = 16 R	Not tested	NDM-1; OXA-10; CMY-4	n.a.
***P. stuartii***	23–11-2015	Urine	>16 R	> = 16 R	R	R	R	R	S	> = 16 R	Not tested	NDM-1; OXA-10; CMY-4	n.a.
***P. stuartii***	30–11-2015	Perineum	2 S	> = 16 R	R	R	R	R	S	> = 16 R	Not tested	NDM-1; OXA-10; CMY-4	n.a.
***P. aeruginosa***	7–11-2015	Throat	2 S	2 S	R	R	Not tested	R	R	0.5 S	Not tested	None detected	ST 235
***P. aeruginosa***	23–11-2015	Throat	4 I	1 S	R	R	Not tested	R	R	<=0.5 S	Not tested	None detected	ST 235
***P. aeruginosa***	30–11-2015	Left dorsum hand	3 I	2 S	R	R	Not tested	R	R	<=0.5 S	Not tested	None detected	ST 235

*ak* amikacin, *gn* gentamicin, *sxt* trimethoprim/sulfamethoxazole, *cip* ciprofloxacin, *fos* fosfomycin, *tig* tigecycline, *col.* colistine, *n.a.* not available

^a^Acquired beta-lactamase genes are presented

**Fig. 1:**
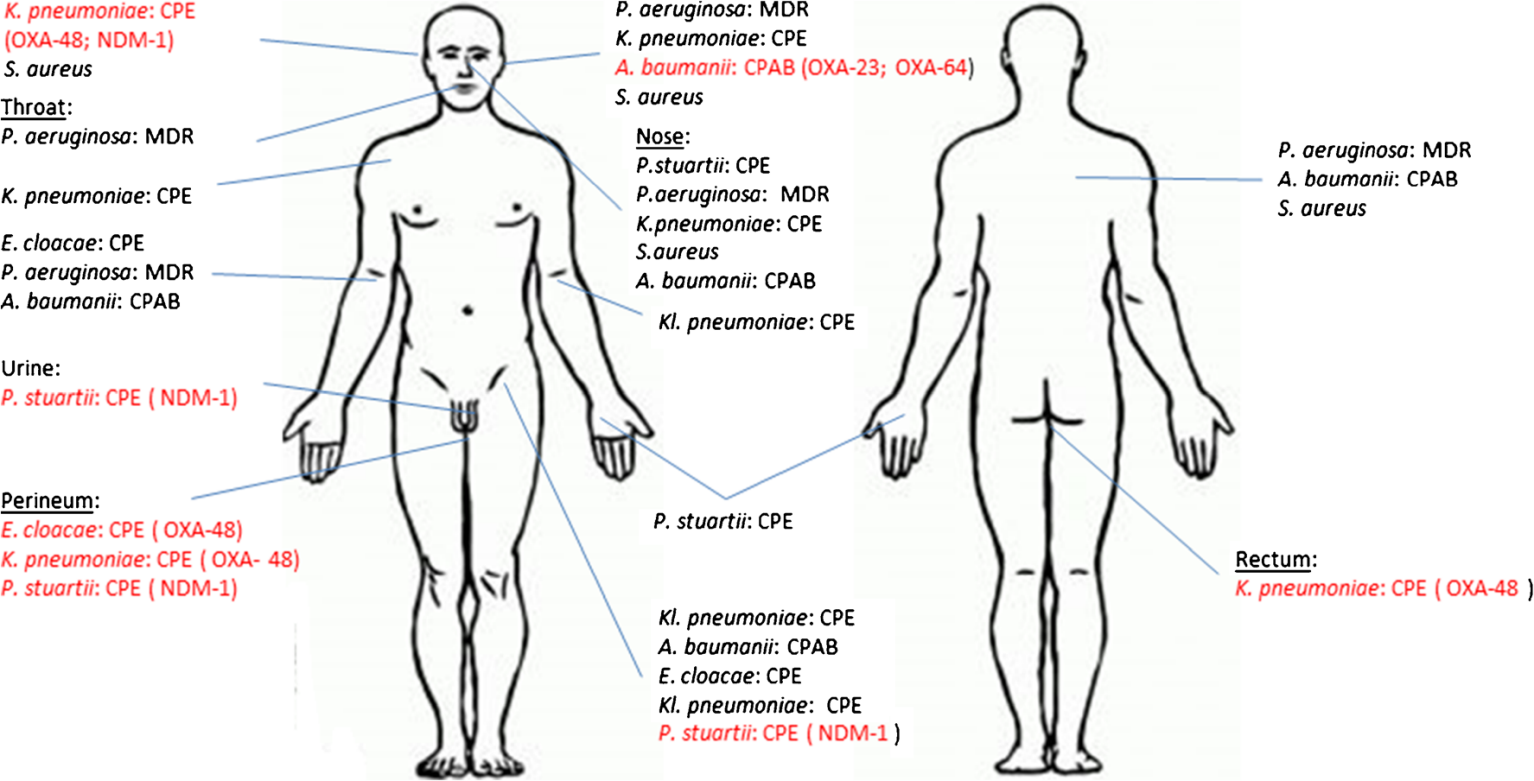
Overview of all body locations and isolated HRGNs. *CPE* Carbapenemase producing Enterobacteriacae, *CPAB* Carbapenemase producing Acinetobacter baumanii, *MDR* Multidrugresistant

An overview of all body locations and isolated HRGNs is presented in Fig. 1. Screening cultures for carriage of HRGNs were positive in nose (4 different isolates), perineum (3 different isolates), rectum (1 isolate) and throat (1 isolate). Cultures from the wound sites showed varying colonization with HRGNs. All sampled wound sites were colonized by HRGNs. The highest number of HRGNs 5/6 were isolated from the groin wound, a donor site wound after the grafting procedures done in Romania. The MDR *Pseudomonas aeruginosa* isolates were exclusively detected in samples from the upper body. We observed differences in colonization in similar body regions: the right forearm was positive for single isolates of highly-resistant *Enterobacter cloacae*, *Pseudomonas aeruginosa*, and *Acinetobacter baumanii*, whereas the left forearm sample grew *Klebsiella pneumoniae*. The matching culture results between urine and both hands are remarkable: in all three samples the NDM-1-producing *Providentia stuartii* were cultured.

All isolates tested resistant to cotrimoxazol, ciprofloxacin and aminogycosides, except for *Klebsiella pneumoniae* isolates of ST 395 and OXA-48-producing *Enterobacter cloacae* which were susceptible to amikacin. Only the NDM-1 producing *Providencia stuartii* NDM-1 and *Klebsiella pneumoniae* ST type 147 were susceptible to fosfomycin. Two colistin-resistant isolates were detected: an OXA-48-producing *Providencia stuartii*, which is intrinsically resistant, and an NDM-1-producing *Klebsiella pneumoniae*.

In addition, multiple carbapenem-non-susceptible *Pseudomonas aeruginosa* isolates of MLST ST235 were grown. The *Pseudomonas* isolates were multidrug resistant, testing resistant to antipseudomonal beta-lactams, fluorquinolones, and aminoglycosides, and susceptible to colistin.

### Review

In our review search we found 84 reports on CPMOs in burn wound care. Of these, 38 were off-topic, thus we included 46 reports. To assess the epidemiology, we present a country by country overview (Table 2) of reports on CPMOs in burn care centers. The country of initial care is shown.

**Table 2:** Review of reported carbapenem-resistant bacterial species isolated from burn patients with the country of initial care

Country	Study	Species	Carbapenemase
Afghanistan	[16]	*P. stuartii*	NDM-1
	[16]	*P. aeruginosa*	VIM-1
Algeria	[17, 18]	*P. aeruginosa*	NDM-1; VIM-4
	[18, 19]	*A. baumanii*	OXA-23
Bulgaria	[20]	*P. aeruginosa*	n.r.
	[20]	*A. baumanii*	n.r.
Brazil	[21]	*P. aeruginosa*	n.r.
China	[22–24]	*P. aeruginosa*	IMP-4; VIM-2
	[24, 25]	*A. baumanii*	OXA-23
	[24]	*K. pneumoniae*	n.r.
Egypt	[26]	*A. baumanii*	n.r.
France	[27]	*A. baumanii*	OXA-58
India	[28]	*K. pneumoniae*	OXA-48&NDM
	[29, 30]	*P. aeruginosa*	n.r.
	[30]	*A. baumanii*	n.r.
Iran	[31–41]	*A. baumanii*	KPC&VIM&OXA-23; VIM&OXA-23; KPC&OXA-23; OXA-23; OXA-40; OXA-23&OXA-40; OXA-23&OXA-58; OXA-23&OXA-40&OXA-58; OXA-40&OXA-58; OXA-23&OXA-58; OXA-143; OXA-58; OXA-23&OXA-24; OXA-24; KPC; VIM
	[41–47]	*P. aeruginosa*	IMP&VIM; IMP; VIM; KPC; AIM
	[41, 48, 49]	*K. pneumoniae*	KPC
Israël	[7]	*P. aeruginosa*	n.r.
	[7]	*A. baumanii*	n.r.
	[7, 50]	*K. pneumoniae*	KPC-3
Italy	[51]	*A. baumanii*	n.r.
Libya	[52]	*A. baumanii*	OXA-23 like; NDM-1
Morocco	[19]	*A.baumanii*	n.r.
Mongolia	[53]	*A. baumanii*	OXA-58
		*P. aeruginosa*	VIM-2
Pakistan	[28, 54]	*K. pneumoniae*	OXA-48&NDM; OXA-48
	[28]	*P. stuartii*	NDM
		*P. aeruginosa*	VIM
		*K. oxytoca*	NDM
		*E. coli*	OXA-48&NDM
		*A. baumanii*	OXA 23
Poland	[55]	*A. baumanii*	OXA-23 like; OXA-40 like
Romania^a^	This study, [28, 56]	*A. baumanii*	OXA-40; OXA-23
	[28]	*E. coli*	OXA-48
	[56]	*P. aeruginosa*	n.r.
	This study, [56]	*K. pneumoniae*	OXA-48&NDM-1; OXA-48
	This study	*P. stuartii*	NDM-1
	This study	*E. cloacae*	OXA-48
Tunisia	[57–59]	*P. aeruginosa*	VIM-2
	[19]	*A.baumanii*	OXA-23 like
Turkey	[60]	*P. aeruginosa*	n.r.
	[60]	*A. baumanii*	n.r.
USA	[61]	*E. cloacae*	KPC-3
	[61, 62]	*K. pneumoniae*	KPC
	[63]	*A. baumanii*	OXA-40

Carbapenemase types/subtypes are shown if tested

*n.r. *no carbapenemase genotyping reported

Some isolates produce multiple carbapenemases. Carbapenemase combinations are noted by “&”

^a^All Romanian studies are on victims of the Colectiv fire disaster

CPMOs in burn patients have been reported from institutions over all continents.

The CPMOs included *Acinetobacter baumanii, Pseudomonas aeroginosa,* and the following Enterobacteriaceae: *Escherichia coli, Klebsiella oxytoca* and *Klebsiella pneumoniae, Enterobacter cloacae,* and *Providencia stuartii.* In these CPMOs, carbapenemase subtypes of KPC, NDM, VIM, IMP and OXA were detected.

The highest number of reports originated from Iran. In 17 publications, patients with burns hospitalized in Iran with carbapenem-resistant *Acinetobacter baumanii* and *Pseudomonas aeruginosa* were reported. The great diversity of carbapenemases detected in *Acinetobacter baumanii* in the Iranian studies is remarkable. One isolate even produced KPC, VIM and OXA-23, representing three different carbapenemases [39].

With our search, we retrieved two studies that described victims of the Colectiv fire disaster from Romania who were treated in England. As in our study, each of them was colonized and or infected with an extraordinary diversity of CPMOs. NDM-producing *Klebsiella pneumoniae*, OXA-48-producing *Klebsiella pneumoniae* and *Escherichia coli*, OXA-40-producing *Acinetobacter baumanii* and carbapenem-resistant *Pseudomonas aeruginosa* were isolated from these patients [13, 14]. The prolonged duration of hospitalization in Romania may have contributed to this extensive colonization with CPMOs. No transfer information had been given to their service about previous microbiology results. One of their patients died of pan-resistant NDM-producing *Klebsiella pneumoniae* septicemia within 48 h of admission. A second case died from severe sepsis due to extensive infected burn injuries: a pan-resistant OXA-48-producing *Klebsiella pneumoniae* grew in the blood culture. Our patient fortunately did not develop infections requiring treatment with systemic antibiotics. Nonetheless, we proactively tested antibiotic susceptibilities for last-line treatment options in all the isolates.

### Recommendations

The Netherlands is a non-endemic country for CPMOs. To maintain this status, we put maximum effort in surveillance and infection control to prevent unnoticed introduction and dissemination of CPMOs. Experience with treatment of burn patients from endemic countries in countries with low CPMO prevalence has been described in six studies [18, 19, 26, 28, 56, 57]. In Table 3, we provide an overview of advice based on these studies completed by recommendations from the present study. All of the studies were alert for the serious risk of CPMO-carriage in transferred patients after hospitalization abroad. Outbreaks with CPMO or outbreak strains or evaluation of contact precautions after an outbreak were described in eight studies [19, 23, 26, 27, 51, 57, 62, 63]. To reduce the risk of transmission of CPMO, patients should be treated in contact isolation in single-patient rooms until culture results are known. Not only can bacteria spread directly by hand contacts [28, 64–66], but also indirectly through the environment and by medical equipment [28, 56, 64–66]. Therefore, we recommend standardized guidelines for the transfer of severely-ill patients between European countries, where detailed procedures on communication, screening and infection prevention measures are described. Especially for specific treatment and in case of international help, clinical staff organizing treatment abroad need to be aware of such guidelines. Furthermore, we recommend education of staff in hand hygiene and isolation precautions, enhancement of disinfection of patient rooms, and single-use of medical equipment if feasible for treatment of burn patients. When transmission of CPMOs is suspected, isolates should be typed and their molecular characteristics should be compared to confirm the clonal spread. Based on this, an outbreak investigation should be started. Control of CPMO or the roll-back of CPMO is today one of the most important goals.

**Table 3:** Recommendations concerning medical microbiology and infection control in treatment of burn wound patients

Recommendations	References
Screening/surveillance of patients on admission (throat, nose, rectum, perineum,) on HRMOs	[64, 65, 67], this study
Sampling of various burn wound sites	This study
Molecular characterization of isolates	This study
Treatment in isolation until cultures are negative for HRMOs	[62, 64, 65, 67]
Proactively testing of antibiotic options	[64, 65], this study
Antimicrobial stewardship/ No systemic antibiotics as prophylaxis	[20, 64, 65, 67], this study
Good communication of the microbiological results	This study
Staff education/ensuring optimal compliance in hand-hygiene and isolation precautions	[20, 28, 62, 64–67]
Enhanced environmental disinfection and environmental sampling following the terminal cleaning	[20, 28, 56, 64–66]
Single use or effective decontamination of medical equipment going from one patient to another	[28]

Samples of throat, nose, rectum, perineum, and all wound sites should be taken at admission to detect all CPMOs and MRSAs carried by the patient. It is important to detect all CPMOs and test their susceptibility patterns, so that targeted therapy can be started in case of systemic infections. Ideally, treating clinicians should already be informed upon patient admission about culture results from the hospital of discharge. For this purpose, good communication within health care networks is needed. This is may be facilitated by the European Burns Association.

To summarize, we showed that burn patients that have been hospitalized in a CPMO endemic country can be colonized by an extensive variety of CPMOs. CPMO presence may differ among body locations, thus we recommend culturing of multiple wound sites. Burn wound colonization by CPMOs is a worldwide problem. There is a high risk for burn patients to develop invasive infections by CPMOs, which require targeted antibiotic therapy. In addition, there is the risk on hospital outbreaks by these CPMOs. Therefore, medical care facilities treating patients with burns transported from endemic regions should have advanced medical microbiology, and infection control systems in place to detect CPMOs, treat infections, and prevent onward transmission.

## References

[1] Cojocariu M, Constanda A (2015) Incendiu in Clubul Colectiv din Bucuresti: 27 de morti, 146 de persoane internate. Marturiile supravietuitorilor si repartizarea ranitilor. Adevarol.ro

[2] Grundmann H, Glasner C, Albiger B, Aanensen DM, Tomlinson CT, Andrasevic AT, Canton R, Carmeli Y, Friedrich AW, Giske CG, Glupczynski Y, Gniadkowski M, Livermore DM, Nordmann P, Poirel L, Rossolini GM, Seifert H, Vatopoulos A, Walsh T, Woodford N, Monnet DL, European Survey of Carbapenemase-Producing Enterobacteriaceae (EuSCAPE) Working Group (2017). Occurrence of carbapenemase-producing Klebsiella pneumoniae and Escherichia coli in the European survey of carbapenemase-producing Enterobacteriaceae (EuSCAPE): A prospective, multinational study. Lancet Infect Dis.

[3] Lixandru BE, Cotar AI, Straut M, Usein CR, Cristea D, Ciontea S, Tatu-Chitoiu D, Codita I, Rafila A, Nica M, Buzea M, Baicus A, Ghita MC, Nistor I, Tuchilus C, Indreas M, Antohe F, Glasner C, Grundmann H, Jasir A, Damian M (2015). Carbapenemase-producing Klebsiella pneumoniae in Romania: a six-month survey. PLoS One.

[4] Wisplinghoff H, Perbix W, Seifert H (1999). Risk factors for nosocomial bloodstream infections due to Acinetobacter baumannii: A case-control study of adult burn patients. Clin Infect Dis.

[5] Alaghehbandan R, Azimi L, Rastegar Lari A (2012). Nosocomial infections among burn patients in Teheran, Iran: A decade later. Ann Burns Fire Disasters.

[6] Taneja N, Chari P, Singh M, Singh G, Biswal M, Sharma M (2013). Evolution of bacterial flora in burn wounds: Key role of environmental disinfection in control of infection. Int J Burns Trauma.

[7] Raz-Pasteur A, Hussein K, Finkelstein R, Ullmann Y, Egozi D (2013). Blood stream infections (BSI) in severe burn patients--early and late BSI: A 9-year study. Burns.

[8] Wang Y, Tang HT, Xia ZF, Zhu SH, Ma B, Wei W, Sun Y, Lv KY (2010). Factors affecting survival in adult patients with massive burns. Burns.

[9] Ugburo AO, Atoyebi OA, Oyeneyin JO, Sowemimo GOA (2004). An evaluation of the role of systemic antibiotic prophylaxis in the control of burn wound infection at the Lagos University teaching hospital. Burns.

[10] European Burns Association (2015) European Practice Guidelines for Burn Care. European Burns Association, The Netherlands

[11] Church D, Elsayed S, Reid O, Winston B, Lindsay R (2006). Burn wound infections. Clin Microbiol Rev.

[12] Patel BM, Paratz JD, Mallet A, Lipman J, Rudd M, Muller MJ, Paterson DL, Roberts JA (2012). Characteristics of bloodstream infections in burn patients: an 11-year retrospective study. Burns.

[13] Kahlmeter G, Brown DFJ, Goldstein FW, MacGowan AP, Mouton JW, Odenholt I, Rodloff A, Soussy C, Steinbakk M, Soriano F, Stetsiouk O (2006). European committee on antimicrobial susceptibility testing (EUCAST) technical notes on antimicrobial susceptibility testing. Clin Microbiol Infect.

[14] Zhou K, Lokate M, Deurenberg RH, Tepper M, Arends JP, Raangs EG, Lo-Ten-Foe J, Grundmann H, Rossen JW, Friedrich AW (2016). Use of whole-genome sequencing to trace, control and characterize the regional expansion of extended-spectrum beta-lactamase producing ST15 Klebsiella pneumoniae. Sci Rep.

[15] Zankari E, Hasman H, Cosentino S, Vestergaard M, Rasmussen S, Lund O, Aarestrup FM, Larsen MV (2012). Identification of acquired antimicrobial resistance genes. J Antimicrob Chemother.

[16] McGann P, Hang J, Clifford RJ, Yang Y, Kwak YI, Kuschner RA, Lesho EP, Waterman PE (2012). Complete sequence of a novel 178-kilobase plasmid carrying bla(NDM-1) in a Providencia stuartii strain isolated in Afghanistan. Antimicrob Agents Chemother.

[17] Meradji S, Barguigua A, Bentakouk MC, Nayme K, Zerouali K, Mazouz D, Chettibi H, Timinouni M (2016). Epidemiology and virulence of VIM-4 metallo-beta-lactamase-producing Pseudomonas Aeruginosa isolated from burn patients in eastern Algeria. Burns.

[18] Moissenet D, Richard P, Granados M, Merens A, Fournier D, Fines-Guyon M, Arlet G, Vu-Thien H (2015). Transfer between an Algerian and a French hospital of four multi-drug resistant bacterial strains together via a single patient. Int J Burns Trauma.

[19] De Vos D, Pirnay JP, Bilocq F, Jennes S, Verbeken G, Rose T, Keersebilck E, Bosmans P, Pieters T, Hing M, Heuninckx W, De Pauw F, Soentjens P, Merabishvili M, Deschaght P, Vaneechoutte M, Bogaerts P, Glupczynski Y, Pot B, van der Reijden TJ, Dijkshoorn L (2016). Molecular epidemiology and clinical impact of Acinetobacter calcoaceticus-baumannii complex in a Belgian burn wound Center. PLoS One.

[20] Leseva M, Arguirova M, Nashev D, Zamfirova E, Hadzhyiski O (2013). Nosocomial infections in burn patients: Etiology, antimicrobial resistance, means to control. Ann Burns Fire Disasters.

[21] de Almeida Silva KC, Calomino MA, Deutsch G, de Castilho SR, de Paula GR, Esper LM, Teixeira LA (2017). Molecular characterization of multidrug-resistant (MDR) Pseudomonas aeruginosa isolated in a burn center. Burns.

[22] Sun FJ, Shi HQ, Zhang XB, Fang YD, Chen YC, Chen JH, Wang Q, Yang B, Feng W, Xia PY (2013). Detection of carbapenemase-encoding genes among clinical isolates of Pseudomonas aeruginosa in a Chinese burn unit. J Burn Care Res.

[23] Zhang R, Mingcheng L, Dong X, Li F (2011). Nosocomial outbreak of carbapenem-resistant Pseudomonas aeruginosa carrying blaVIM-2 in burn wards, China. Braz J Infect Dis.

[24] Cen H, Wu Z, Wang F, Han C (2015). Pathogen distribution and drug resistance in a burn ward: A three-year retrospective analysis of a single center in China. Int J Clin Exp Med.

[25] Huang G, Yin S, Gong Y, Zhao X, Zou L, Jiang B, Dong Z, Chen Y, Chen J, Jin S, Yuan Z, Peng Y (2016). Multilocus sequence typing analysis of Carbapenem-resistant Acinetobacter Baumannii in a Chinese burns institute. Front Microbiol.

[26] Ho AL, Chambers R, Malic C, Papp A (2016) Universal contact precautions do not change the prevalence of antibiotic resistant organisms in a tertiary burn unit. Burns 43(2):265–272 10.1016/j.burns.2016.11.001 27915096

[27] Heritier C, Dubouix A, Poirel L, Marty N, Nordmann P (2005). A nosocomial outbreak of Acinetobacter Baumannii isolates expressing the carbapenem-hydrolysing oxacillinase OXA-58. J Antimicrob Chemother.

[28] Teare L, Myers J, Kirkham A, Tredoux T, Martin R, Boasman S, Wisbey A, Charlton C, Dziewulski P (2016). Prevention and control of carbapenemase-producing organisms at a regional burns centre. J Hosp Infect.

[29] Jena J, Debata NK, Sahoo RK, Subudhi E (2015). Phylogenetic study of metallo-beta-lactamase producing multidrug resistant Pseudomonas Aeruginosa isolates from burn patients. Burns.

[30] Kumar SH, De AS, Baveja SM, Gore MA (2012). Prevalence and risk factors of Metallo beta-lactamase producing Pseudomonas Aeruginosa and Acinetobacter species in burns and surgical wards in a tertiary care hospital. J Lab Physicians.

[31] Adibhesami H, Douraghi M, Zeraati H, Bazmi F, Rahbar M, Pourmand MR, Tabrizi MS, Aliramezani A, Ghourchian S (2016). Carbapenem-resistant Acinetobacter baumannii (CRAB) recovered from burn patients. J Pharm Pharm Sci.

[32] Farshadzadeh Z, Hashemi FB, Rahimi S, Pourakbari B, Esmaeili D, Haghighi MA, Majidpour A, Shojaa S, Rahmani M, Gharesi S, Aziemzadeh M, Bahador A (2015). Wide distribution of carbapenem resistant Acinetobacter Baumannii in burns patients in Iran. Front Microbiol.

[33] Bahador A, Raoo An R, Farshadzadeh Z, Beitollahi L, Khaledi A, Rahimi S, Mokhtaran M, Mehrabi Tavana A, Esmaeili D (2015). The prevalence of IS Aba 1 and IS Aba 4 in Acinetobacter Baumannii species of different international clone lineages among patients with burning in Tehran, Iran. Jundishapur J Microbiol.

[34] Mahdian S, Sadeghifard N, Pakzad I, Ghanbari F, Soroush S, Azimi L, Rastegar-Lari A, Giannouli M, Taherikalani M (2015). Acinetobacter Baumannii clonal lineages I and II harboring different carbapenem-hydrolyzing-beta-lactamase genes are widespread among hospitalized burn patients in Tehran. J Infect Public Health.

[35] Azimi L, Talebi M, Pourshafie MR, Owlia P, Rastegar Lari A (2015). Characterization of Carbapenemases in extensively drug resistance Acinetobacter baumannii in a burn care center in Iran. Int J Mol Cell Med.

[36] Salimizand H, Noori N, Meshkat Z, Ghazvini K, Amel SJ (2015). Prevalence of Acinetobacter Baumannii harboring ISAba1/bla OXA-23-like family in a burn center. Burns.

[37] Nasrolahei M, Zahedi B, Bahador A, Saghi H, Kholdi S, Jalalvand N, Esmaeili D (2014). Distribution of bla(OXA-23), ISAba , Aminoglycosides resistant genes among burned & ICU patients in Tehran and Sari, Iran. Ann Clin Microbiol Antimicrob.

[38] Pajand O, Rezaee MA, Nahaei MR, Mahdian R, Aghazadeh M, Soroush MH, Tabrizi MS, Hojabri Z (2013). Study of the carbapenem resistance mechanisms in clinical isolates of Acinetobacter baumannii: Comparison of burn and non-burn strains. Burns.

[39] Azimi L, Talebi M, Khodaei F, Najafi M, Lari AR (2016). Comparison of multiple-locus variable-number tandem-repeat analysis with pulsed-field gel electrophoresis typing of carbapenemases producing Acinetobacter baumannii isolated from burn patients. Burns.

[40] Azimi L, Lari AR, Talebi M, Owlia P, Alaghehbandan R, Asghari B, Lari ER (2015). Inhibitory-based method for detection of Klebsiella Pneumoniae carbapenemase Acinetobacter baumannii isolated from burn patients. Indian J Pathol Microbiol.

[41] Azimi L, Rastegar Lari A, Alaghehbandan R, Alinejad F, Mohammadpoor M, Rahbar M (2012). KPC-producer gram negative bacteria among burned infants in Motahari hospital, Tehran: First report from Iran. Ann Burns Fire Disasters.

[42] Radan M, Moniri R, Khorshidi A, Gilasi H, Norouzi Z, Beigi F, Dasteh Goli Y (2016). Emerging Carbapenem-resistant Pseudomonas aeruginosa isolates carrying blaIMP among burn patients in Isfahan, Iran. Arch Trauma Res.

[43] Farajzadeh Sheikh A, Rostami S, Jolodar A, Tabatabaiefar MA, Khorvash F, Saki A, Shoja S, Sheikhi R (2014). Detection of metallo-beta lactamases among carbapenem-resistant Pseudomonas aeruginosa. Jundishapur J Microbiol.

[44] Salimi F, Eftekhar F (2014). Prevalence of blaIMP, and blaVIM gene carriage in metallo-beta-lactamase-producing burn isolates of Pseudomonas aeruginosa in Tehran. Turk J Med Sci.

[45] Neyestanaki DK, Mirsalehian A, Rezagholizadeh F, Jabalameli F, Taherikalani M, Emaneini M (2014). Determination of extended spectrum beta-lactamases, metallo-beta-lactamases and AmpC-beta-lactamases among carbapenem resistant Pseudomonas Aeruginosa isolated from burn patients. Burns.

[46] Lari AR, Azimi L, Rahbar M, Alaghehbandan R, Sattarzadeh-Tabrizi M (2014) First report of Klebsiella pneumonia carbapenemase-producing Pseudomonas aeruginosa isolated from burn patients in Iran: phenotypic and genotypic methods. GMS Hyg Infect Control 9:Doc06 10.3205/dgkh000226 PMC3960934 24653970

[47] Yousefi S, Nahaei M, Farajnia S, Ghojazadeh M, Akhi M, Sharifi Y, Milani M, Ghotaslou R (2010). Class 1 integron and Imipenem resistance in clinical isolates of Pseudomonas aeruginosa: Prevalence and antibiotic susceptibility. Iran J Microbiol.

[48] Eftekhar F, Naseh Z (2015). Extended-spectrum beta-lactamase and carbapenemase production among burn and non-burn clinical isolates of Klebsiella Pneumoniae. Iran J Microbiol.

[49] Rastegar Lari A, Azimi L, Rahbar M, Fallah F, Alaghehbandan R (2013). Phenotypic detection of Klebsiella Pneumoniae carbapenemase among burns patients: First report from Iran. Burns.

[50] Benenson S, Navon-Venezia S, Carmeli Y, Adler A, Strahilevitz J, Moses AE, Block C (2009). Carbapenem-resistant Klebsiella Pneumoniae endocarditis in a young adult. Successful treatment with gentamicin and colistin. Int J Infect Dis.

[51] Casini B, Selvi C, Cristina ML, Totaro M, Costa AL, Valentini P, Barnini S, Baggiani A, Tagliaferri E, Privitera G (2017). Evaluation of a modified cleaning procedure in the prevention of carbapenem-resistant Acinetobacter Baumannii clonal spread in a burn intensive care unit using a high-sensitivity luminometer. J Hosp Infect.

[52] Mathlouthi N, El Salabi AA, Ben Jomaa-Jemili M, Bakour S, Al-Bayssari C, Zorgani AA, Kraiema A, Elahmer O, Okdah L, Rolain JM, Chouchani C (2016). Early detection of metallo-beta-lactamase NDM-1- and OXA-23 carbapenemase-producing Acinetobacter Baumannii in Libyan hospitals. Int J Antimicrob Agents.

[53] Baljin B, Baldan G, Chimeddorj B, Tulgaa K, Gunchin B, Sandag T, Pfeffer K, MacKenzie CR, Wendel AF (2016). Faecal carriage of gram-negative multidrug-resistant bacteria among patients hospitalized in two centres in Ulaanbaatar, Mongolia. PLoS One.

[54] Sepehri S, Poliquin G, Alfattoh N, Boyd D, Mulvey M, Denisuik A, Fanella S, Karlowsky J, Walkty A (2014). Osteomyelitis due to multiple carbapenemase-producing gram-negative bacteria: the first case report of a GES-13-producing Pseudomonas aeruginosa isolate in Canada. Can J Infect Dis Med Microbiol.

[55] Nowak P, Paluchowska P, Budak A (2012). Distribution of blaOXA genes among carbapenem-resistant Acinetobacter Baumannii nosocomial strains in Poland. New Microbiol.

[56] Garvey MI, Bradley CW, Jumaa P (2016). Environmental decontamination following occupancy of a burns patient with multiple carbapenemase-producing organisms. J Hosp Infect.

[57] Maillet M, Pelloux I, Forli A, Vancoetsem K, Cheong Sing JS, Marfaing S, Ducki S, Batailler P, Mallaret MR (2014). Nosocomial transmission of carbapenem-resistant Pseudomonas Aeruginosa among burn patients. Infect Control Hosp Epidemiol.

[58] Belotti PT, Thabet L, Laffargue A, Andre C, Coulange-Mayonnove L, Arpin C, Messadi A, M'Zali F, Quentin C, Dubois V (2015). Description of an original integron encompassing blaVIM-2, qnrVC1 and genes encoding bacterial group II intron proteins in Pseudomonas Aeruginosa. J Antimicrob Chemother.

[59] Zoghlami A, Kanzari L, Boukadida J, Messadi AA, Ghanem A (2012). Epidemiological profile and antibiotic resistance of Pseudomonas aeruginosa isolates in burn and traumatology center in Tunisia over a three-year period. Tunis Med.

[60] Altoparlak U, Aktas F, Celebi D, Ozkurt Z, Akcay MN (2005). Prevalence of metallo-beta-lactamase among Pseudomonas aeruginosa and Acinetobacter baumannii isolated from burn wounds and in vitro activities of antibiotic combinations against these isolates. Burns.

[61] Kanamori H, Parobek CM, Juliano JJ, van Duin D, Cairns BA, Weber DJ, Rutala WA (2017) A prolonged outbreak of KPC-3-producing *Enterobacter cloacae* and *Klebsiella pneumoniae* driven by multiple mechanisms of resistance transmission at a large academic burn center. Antimicrob Agents Chemother 61:16 10.1128/AAC.01516-16 PMC5278681 27919898

[62] Rosenberger LH, Hranjec T, Politano AD, Swenson BR, Metzger R, Bonatti H, Sawyer RG (2011). Effective cohorting and "superisolation" in a single intensive care unit in response to an outbreak of diverse multi-drug-resistant organisms. Surg Infect.

[63] Munoz-Price L, Zembower T, Penugonda S, Schreckenberger P, Lavin MA, Welbel S, Vais D, Baig M, Mohapatra S, Quinn JP, Weinstein RA (2010). Clinical outcomes of carbapenem-resistant Acinetobacter baumannii bloodstream infections: Study of a 2-state monoclonal outbreak. Infect Control Hosp Epidemiol.

[64] French CE, Coope C, Conway L, Higgins JP, McCulloch J, Okoli G, Patel BC, Oliver I (2017). Control of carbapenemase-producing Enterobacteriaceae outbreaks in acute settings: An evidence review. J Hosp Infect.

[65] Borer A, Eskira S, Nativ R, Saidel-Odes L, Riesenberg K, Livshiz-Riven I, Schlaeffer F, Sherf M, Peled N (2011). A multifaceted intervention strategy for eradication of a hospital-wide outbreak caused by carbapenem-resistant Klebsiella pneumoniae in southern Israel. Infect Control Hosp Epidemiol.

[66] Enfield KB, Huq NN, Gosseling MF, Low DJ, Hazen KC, Toney DM, Slitt G, Zapata HJ, Cox HL, Lewis JD, Kundzins JR, Mathers AJ, Sifri CD (2014). Control of simultaneous outbreaks of carbapenemase-producing enterobacteriaceae and extensively drug-resistant Acinetobacter baumannii infection in an intensive care unit using interventions promoted in the Centers for Disease Control and Prevention 2012 carbapenemase-resistant Enterobacteriaceae toolkit. Infect Control Hosp Epidemiol.

[67] Merchant N, Smith K, Jeschke MG (2015). An ounce of prevention saves tons of lives: infection in burns. Surg Infect.

